# Editorial: Non-invasive neuromodulation for movement and emotional disorders

**DOI:** 10.3389/fpsyg.2022.1079531

**Published:** 2022-11-23

**Authors:** Junjie Bu, Huijing Huang

**Affiliations:** ^1^School of Biomedical Engineering, Anhui Medical University, Hefei, China; ^2^School of Mental Health and Psychological Sciences, Anhui Medical University, Hefei, China

**Keywords:** movement disorders, emotional disorders, non-invasive neuromodulation, neurofeedback, transcranial current stimulation, transcranial magnetic stimulation

This topic aims at collecting more articles about non-invasive neuromodulation techniques toward movement or emotional disorders, especially using novel neuromodulation, providing a platform for researchers to overview the newest findings and promoting the optimization and clinical application of non-invasive neuromodulation techniques toward movement or emotional disorders. And we hope these researches are able to shed light on but not limited to the following subjects: demonstrating the neural mechanisms of movement or emotional disorders, verifying the efficacy of non-invasive neuromodulation efficacy toward movement or emotional disorders, and revealing the potential use of the combination of neuroimaging measures and non-invasive neuromodulation. Currently, our topic publishes 4 original research papers. Two of them focused on exploring the neural mechanisms of Alzheimer's disease and Alcohol use disorder by non-invasive technologies. One illustrated the interplay of movement and emotion. And the last one examined the efficacy of non-invasive neuromodulation technologies toward depression.

Movement and emotional disorders have been global problems for a long time. Diverse researches that focus on the mechanisms and treatments of movement or emotional disorders in human subjects have been accessed (Edwards and Bhatia, 2012; Barlow et al., 2017). As technology advances, numerous neuroimaging studies have identified the special changes in some movement or emotion disorders (Sasikumar and Strafella, 2021; Shackman and Fox, 2021), based on which non-invasive neuromodulation technologies, a rapidly developing and highly multidisciplinary field, might guide a novel direction in the non-drug treatment.

Learning burnout refers to negative attitudes and behaviors that lead to psychological and behavioral problems, such as anxiety, depression, eating disorders and so on. Yu et al. found that school psychological environment could directly influence medical students' learning burnout, leading different study performances in medical students. Improving students' internal emotional factors helps reduce learning burnout (i.e., self-esteem, sense of identity and belonging to the school, etc.).

With relentless refinement, more non-invasive technologies are available in movement and emotional disorders. Alzheimer's disease (AD) is a typical disease resulting in severe mental dysfunctions. Miao et al. analyzed T1-weighted anatomical images and gray matter volume of matched AD, mild cognitive impairment (MCI), and healthy controls (HCs). The final results reveal distinct patterns of morphological atrophy in AD and MCI, providing new insights into pathology of AD. In addition, they identified altered structural covariance network of hippocampus in AD compared with HCs in reward-related brain regions, which might be related to apathy in AD. Alcohol use disorder (AUD) is one of the most common substance use disorders contributing to both behavioral and cognitive impairments in patients. Duan et al. adopted a newly proposed method named functional connectivity density (FCD) to depict altered voxel-wise functional coordination in AUD. It turned out that patients with AUD exhibited significantly altered functional connectivity patterns mainly in several left hemisphere brain regions, while patients with AUD cognitive impairment would suffer less severe alcohol dependence. The specific pattern and difference they found provided insight into possible pathological mechanisms.

Non-invasive neuromodulation technologies have proven its efficacy in certain movement and emotional disorders. Major depression is a kind of emotional dysfunction that could eventually lead to physical detriments. Du et al. developed a scalp landmark-based approach to locate the two symptom-specific targets for depressive patients at group level by using rTMS, which would greatly facilitate the application of these novel findings in clinical treatment. However, the accuracy was highly varied at individual level and further improvement is needed.

Neuroimaging measures lay the visualized research foundation and offer an entry point for further and deeper studies. And non-invasive neuromodulation techniques, have shown incredible influence on movement and emotional disorders, helping us figure out the neural mechanisms and even serving as treatments (Ganguly et al., 2020; Yamada et al., 2022). Together, their combination is an area with great potential. However, it still entails massive researches and experiments to optimize and prove its feasibility, universality and safety in the future.

This Research Topic will enable novel developments of non-invasive neuromodulation, as well as extensive validation studies of current and future non-invasive neuromodulation with neuroimaging measures.

## Author contributions

All authors listed have made a substantial, direct, and intellectual contribution to the work and approved it for publication.

## Funding

JB was supported by Natural Science Foundation of China (32000750), Anhui Province Nature Science Foundation (2008085QH369 and 2108085MH303), Basic and Clinical Collaborative Research Improvement Project of Anhui Medical University (2020xkjT020), and Scientific Research Improvement Project of Anhui Medical University (2021xkjT018).

## Conflict of interest

The authors declare that the research was conducted in the absence of any commercial or financial relationships that could be construed as a potential conflict of interest.

## Publisher's note

All claims expressed in this article are solely those of the authors and do not necessarily represent those of their affiliated organizations, or those of the publisher, the editors and the reviewers. Any product that may be evaluated in this article, or claim that may be made by its manufacturer, is not guaranteed or endorsed by the publisher.
